# Low expression of miR-29a is associated with aggressive biology and worse survival in gastric cancer

**DOI:** 10.1038/s41598-021-93681-z

**Published:** 2021-07-08

**Authors:** Yoshihisa Tokumaru, Masanori Oshi, Michelle R. Huyser, Li Yan, Masahiro Fukada, Nobuhisa Matsuhashi, Manabu Futamura, Yukihiro Akao, Kazuhiro Yoshida, Kazuaki Takabe

**Affiliations:** 1 Breast Surgery, Department of Surgical Oncology, Roswell Park Comprehensive Cancer Center, Elm and Carlton Streets, Buffalo, NY 14263 USA; 2 Department of Surgical Oncology, Graduate School of Medicine, Gifu University, 1-1 Yanagido, Gifu, 501-1194 Japan; 3 Department of Gastroenterological Surgery, Yokohama City University Graduate School of Medicine, Yokohama, 236-0004 Japan; 4 Department of Biostatistics and Bioinformatics, Roswell Park Comprehensive Cancer Center, Buffalo, NY 14263 USA; 5 United Graduate School of Drug and Medical Information Sciences, Gifu University, 1-1 Yanagido, Gifu, 501-1194 Japan; 6 Department of Surgery, Niigata University Graduate School of Medical and Dental Sciences, Niigata, 951-8510 Japan; 7 Department of Surgery, University at Buffalo Jacobs School of Medicine and Biomedical Sciences, The State University of New York, Buffalo, NY 14263 USA; 8 Department of Breast Oncology and Surgery, Tokyo Medical University, 6-7-1 Nishishinjuku, Shinjuku, Tokyo, 160-8402 Japan; 9 Department of Breast Surgery, Fukushima Medical University School of Medicine, Fukushima, 960-1295 Japan

**Keywords:** Tumour biomarkers, miRNAs

## Abstract

Advanced gastric cancer (GC) is one of the most lethal cancer types, thus a better understanding of its biology in patients is urgently needed. MicroRNA (miR)-29a is a known tumor suppressive miR that is related to metastasis, but its clinical relevance in GC remains ambiguous. Here, using a large GC patient cohort we hypothesized that low expression of miR-29a in GC is associated with aggressive cancer biology and worse survival. We demonstrated that low miR-29a GC enriched cell proliferation, apoptosis, metastasis, and angiogenesis related gene sets, as well as the higher expression of related genes. Low miR-29a GC was associated with less anti-cancer immune cell infiltration as well as immune related scoring. Low miR-29a GC demonstrated a worse overall survival (OS) as well as disease specific survival (DSS) compared with high expressing miR-29a GC. Notably, low miR-29a expression was the only factor, other than residual tumor status, to be an independent prognostic biomarker of worse OS and DSS. In conclusion, low miR-29a GC was associated with aggressive cancer biology and worse OS as well as DSS. Additionally, low expression of miR-29a was an independent prognostic biomarker of OS and DSS in gastric cancer patients.

## Introduction

Gastric cancer (GC) is one of the leading causes of cancer related deaths worldwide^1,2^. Although multimodality treatment approaches have improved survival outcomes of GC patients, there is still room for improvement, especially regarding advanced stage GC patients. Thus, there is a need to develop, or discover, an informative prognostic biomarker for GC.

MicroRNA (miRNA) is one of the epigenetic regulators of gene expression for specific genes associated with cancer progression, such as cell proliferation, cell invasion, and metastasis in numerous cancers, including gastric cancer^3–5^. Some miRNAs function as tumor suppressive miRNA by inhibiting *c-MYC*, one of the most studied oncogenes that promote cancer cell proliferation^6,7^, such as miR-429^8^ and miR-494^9^. Metastasis contributes to the higher mortality of GC patients. One of the key signaling pathways that promotes metastasis is the Wnt/β-catenin pathway^10^. This pathway is regulated by several miRNAs in GC, such as miR-204^11^ and miR-491-5p^12^.

Another miRNA associated with metastasis is miR-29a^13–15^. MiR-29a suppresses metastasis by inhibiting metastasis-related genes, such as vascular endothelial growth factor A (*VEGF-A*)^13^, telomerase reverse transcriptase (*TERT*), integrinβ1 (*ITGB1*)^14^, and Roundabout homolog 1 (*ROBO1*)^15^ in vitro and/or in vivo in gastric cancer. MiR-29a also has been reported to be associated with cell proliferation^16,17^ and angiogenesis^18^. In one study, miR-29a inhibits cell proliferation of GC cells by targeting the APC domain containing 2 (*p42.3* or *SAPCD2*) in vitro and in vivo. Furthermore, the expression level of miR-29a had negative correlation with the *p42.3* expression in 60 clinical patient samples^16^. According to Zhao et al., cyclin dependent kinase 2 (*CDK 2*), *CDK4*, and *CDK6* are the target of miR-29a and miR-29a, which inhibited the cell proliferation by those genes in vitro^17^. Zhang et al. also reported that miR-29a suppressed angiogenesis in GC by targeting *VEGF* expression in vitro and in vivo^18^.

Because the previous studies introduced above, are mainly pre-clinical studies using cells and animal models or included very small numbers of clinical samples, the clinical relevance of miR-29a expression in GC patients remains unclear. Recently, translational research utilizing bioinformatic tools such as xCell^19^ and gene set enrichment analysis (GSEA)^20^ has gained popularity. Our group has been aggressively pursuing such translational research to elucidate the clinical significance of microRNAs in breast cancer^21–27^. For instance, we demonstrated that low miR-34a expressing tumors were associated with less aggressive cancer biology^26^, and that high expressing miR-342 tumors were associated with better overall survival^27^.

Whether the results of preclinical studies can directly translate into the clinical setting is unknown until it is investigated in large patient cohorts. In the current study, we aimed to elucidate the clinical relevance of miR-29a expression levels in GC patients utilizing a large publicly available cohort, The Cancer Genome Atlas (TCGA). We hypothesized that low expressing miR-29a GC is associated with aggressive GC biology including cell proliferation, angiogenesis, and metastasis, as well as worse prognosis.

## Materials and methods

### Data acquisition of gastric cancer patients in TCGA

We acquired gastric cancer patients’ clinicopathological and transcriptomic data in The Cancer Genome Atlas Stomach Cancer (TCGA-STAD) through cBioPortal as previously described^28–30^. We also obtained the microRNA expression data through Broad Institute Firehose (http://gdac.broadinstitute.org/) as described previously^24,31,32^. A total of 335 gastric cancer patients with both miR-29a and transcriptomic data were included in this study. Given that TCGA is publicly accessible and deidentified database, the ethics of our institution waived Institutional Review Board for the current study.

### Gene set enrichment analysis (GSEA)

Broad Institute (http://software.broadinstitute.org/gsea/index.jsp) has developed and maintained a publicly available software, Gene set enrichment analysis (GSEA)^20^. We utilized the MSigDB Hallmark collection gene sets, which demonstrate the well-known cancer biological states as reported previously^33–35^. The statistical significance was defined as false discovery rate (FDR) < 0.25, as the developer of GSEA has recommended.

### The estimation of immune cell composition within the tumor immune microenvironment and immune related scores

To investigate the tumor immune microenvironment, we used a computational algorithm, xCell, which was reported by Aran et al.^19^. This method estimates the composition of 64 types of immune and stromal cells utilizing gene expression data of a bulk tumor. Utilizing this method, our group reported the clinical relevance of immune cells^36–39^, stromal cells^40,41^, and tumor immune microenvironment^42–54^ in a number of settings in multiple types of cancer. The data for the current study was obtained through the webpage of xCell (https://xcell.ucsf.edu/).

### Predicted target genes of miR-29a

We utilized the publicly accessible website, miRDB (http://mirdb.org), to obtain the top 30 predicted target genes of miR-29a^55^.

### Others

All the statistical analysis of the present study was performed by R software (http://www.r-project.org/) (version 4.0.2). Survival analysis was performed by plotting Kaplan–Meier curve with log rank test. We utilized one-way ANOVA for the comparison of multiple groups. The Fisher’s exact test was utilized for the comparison of two groups. A two-sided p < 0.05 was considered statistically significant. The black line inside boxplots (Tukey type) demonstrates median value and the span of rectangle demonstrates inter-quartile ranges.

## Results

### Low expressing miR-29a gastric cancer (GC) enriched the gene sets related to cell proliferation and apoptosis, but were not associated with MKI67 expression and pathological grade

**Figure 1:**
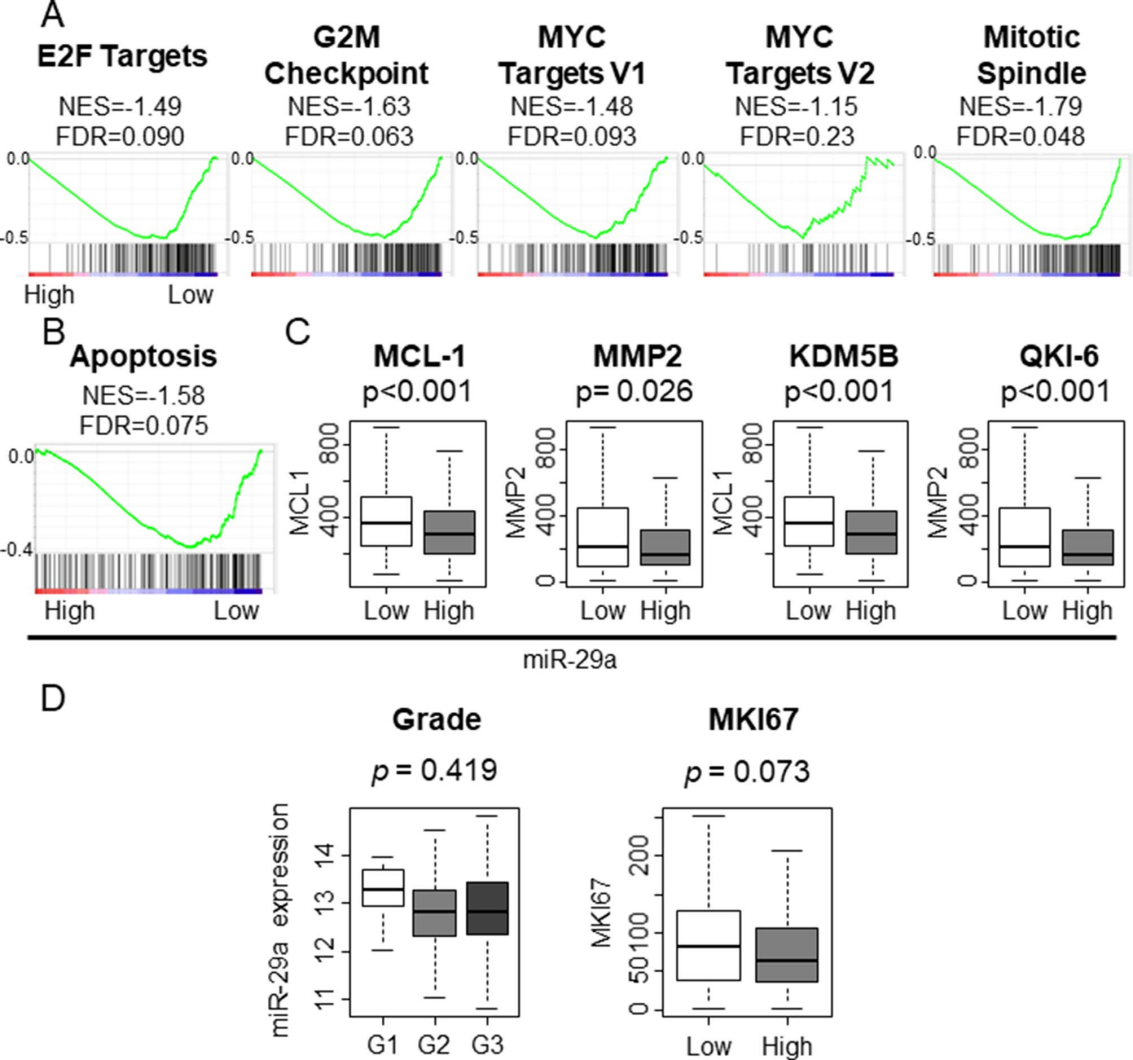
Association between miR-29a and pathological grade or *MKI67* expression and gene set enrichment analysis (GSEA) in TCGA STAD cohort. (**A**) GSEA of cell proliferation-related gene sets. (**B**) GSEA of apoptosis-related gene sets. (**C**) The association between miR-29a and apoptosis-inducing genes. *KDM5B* lysine demethylation 5B, *MCL-1* myeloid cell leukemia 1, *MMP2* matrix metalloproteinase 2, *QKI-6* Quaking gene isoform 6, *NES* normalized enrichment score, *FDR* false discovery rate. The statistical significance was defined by *p* < 0.05 or FDR < 0.25. Tukey-type boxplots demonstrate median and inter-quartile level values. (**D**) The association between miR-29a and pathological grade or *MKI67* expression in TCGA cohort. Open box, shaded box, Closed box demonstrate Grade1, Grade 2, and Grade 3, respectively. The median cutoff of miR-29a was used to divide the group into two groups: high and low expression groups.

Previous in vitro studies reported that tumor suppressive miR-29a inhibited cell proliferation in gastric cancer (GC)^16,17^. To this end, we hypothesized that lower expression of miR-29a in GC would translate into more aggressive cancer biology. To test our hypothesis, we performed gene set enrichment analysis (GSEA), which revealed that low expressing miR-29a GC enriched all of cell proliferation-related Hallmark gene sets including E2F Targets, G2M Checkpoint, MYC Targets V1, MYC Targets V2, and Mitotic Spindle (Fig. 1A; normalized enrichment score (NES) = − 1.49 false discovery rate (FDR) = 0.090, NES = − 1.63 FDR = 0.063, NES = − 1.48 FDR = 0.093, NES = − 1.15 FDR = 0.23, and NES = − 1.79 FDR = 0.048 respectively). Given that low miR-29a GC is associated with high cell proliferation, we expected those tumors to be associated with increased cell death. As expected, low miR-29a GC enriched the gene set related to apoptosis (Fig. 1B; NES = − 1.58 FDR = 0.075). In order to validate miR-29a expression with apoptosis, we investigated the expression of genes that are well-known to be related with apoptosis. Myeloid cell leukemia 1 (MCL-1), matrix metalloproteinase 2 (MMP2), lysine demethylation 5B (KDM5B), and Quaking gene isoform 6 (QKI-6) genes were all consistently and significantly elevated in miR-29c low tumors (Fig. 1C; p < 0.001, p = 0.026, p < 0.001, and p < 0.001, respectively). We then investigated how miR-29a levels translate to clinical features. Nottingham pathological grades and *MKI67* expression are most commonly used as markers of cell proliferation in the clinical setting, but we found no association between pathological grades and *MKI67* expression as well as miR-29a expression levels (Fig. 1D). These findings demonstrated that low miR-29a expressing GC was associated with high cell proliferation and apoptosis gene expression signatures, but did not translate into clinical features, such as pathological grade and *MKI67* expression levels.

### Low miR-29a GC enriched metastasis related gene sets and was associated with a higher expression level of metastasis promoting genes

**Figure 2:**
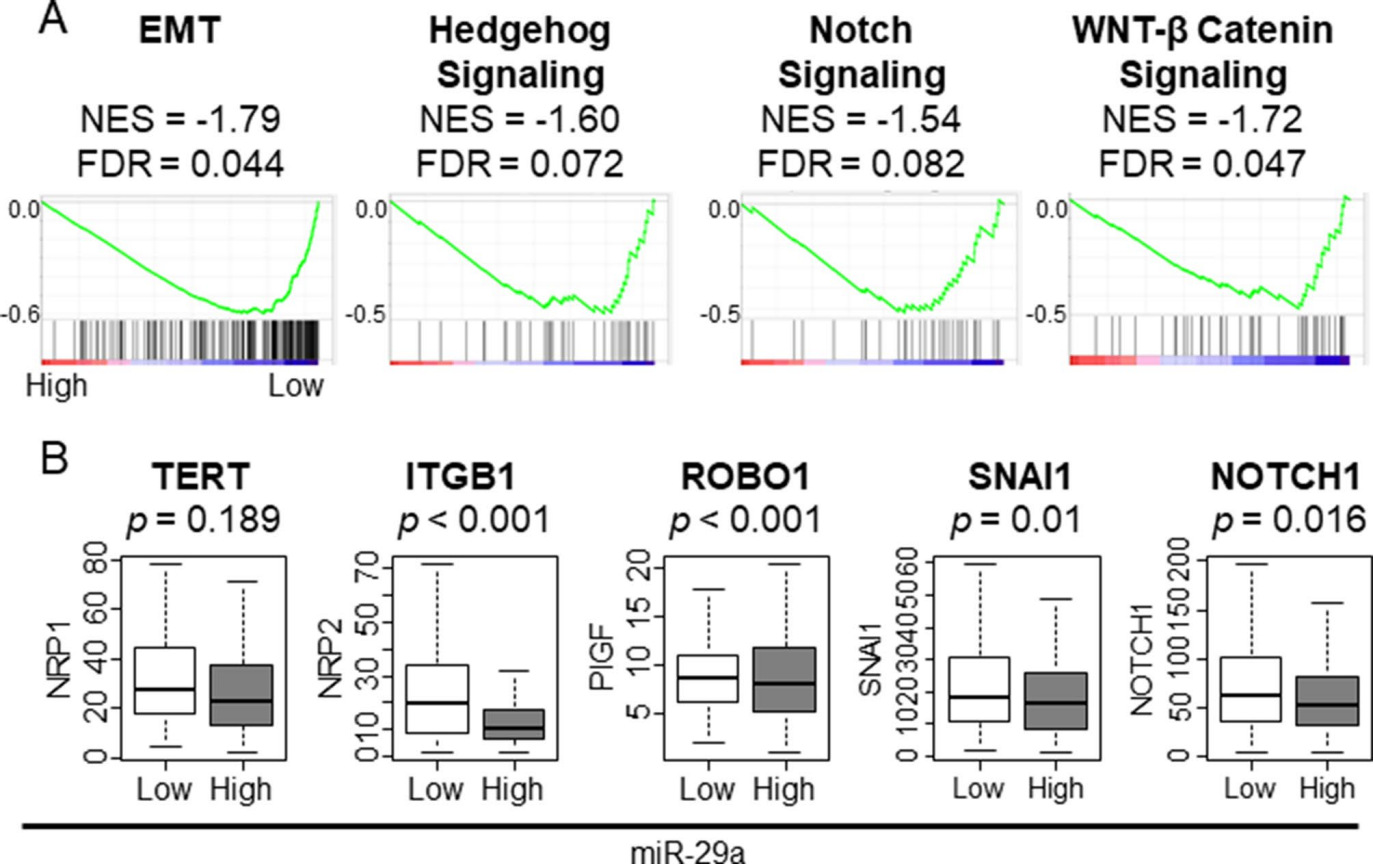
GSEA of metastasis-related gene sets and the association between miR-29a and metastasis-related genes in TCGA STAD cohort. (**A**) GSEA of cell metastasis-related gene sets. (**B**) The association between miR-29a and metastasis-promoting genes. *EMT* Epithelial–mesenchymal transition, *ITGB1* integrinβ1, *NOTCH1* notch receptor 1, *SNAI1* snail family transcriptional repressor 1, *TERT* telomerase reverse transcriptase, *NES* normalized enrichment score, *FDR* false discovery rate. The statistical significance was defined by *p* < 0.05 or FDR < 0.25. Tukey-type boxplots demonstrate median and inter-quartile level values.

Since highly proliferative tumors tend to have a higher risk of metastasis, we expected that low miR-29a GC may have higher tendency to metastasize. GSEA revealed that low expressing miR-29a GC enriched metastasis-related gene sets, such as EMT, Hedgehog Signaling, Notch Signaling, WNT-β Catenin Signaling (Fig. 2A; NES = − 1.79 FDR = 0.044, NES = − 1.60 FDR = 0.072, NES = − 1.54 FDR = 0.082, and NES = − 1.72 FDR = 0.047, respectively). Furthermore, we found that previously reported gene expressions known to promote cancer cell invasion and metastasis such as telomerase reverse transcriptase (*TERT*), integrinβ1 (*ITGB1*), Roundabout homolog 1 (*ROBO1*), snail family transcriptional repressor 1 (*SNAI1*), and notch receptor 1 (*NOTCH1*) were all significantly lower in low miR-29a expressing tumors, except for *TERT* (Fig. 2B; p = 0.189, p < 0.001, p < 0.001, p = 0.01, and p = 0.016, respectively). These results demonstrate that low expressing miR-29a GC is associated with metastasis.

### Low expressing miR-29a GC enriched angiogenesis-related gene sets and was associated with higher expression of the genes contributing to angiogenesis

**Figure 3:**
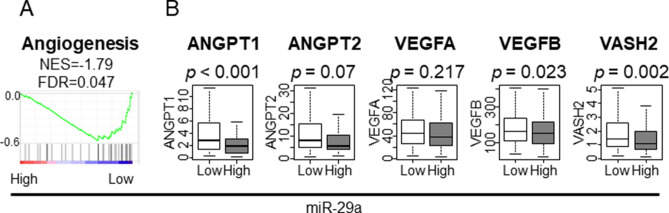
GSEA of angiogenesis-related gene sets and the association between miR-29a and angiogenesis -related genes in TCGA STAD cohort. (**A**) GSEA of cell angiogenesis-related gene sets. (**B**) The association between miR-29a and angiogenesis-promoting genes. *ANGPT1/2* Angiopoietin 1/2, *VASH2* vasohibin 2, *VEGF1/2* vascular endothelial growth factor1/2, *NES* normalized enrichment score, *FDR* false discovery rate. The statistical significance was defined by *p* < 0.05 or FDR < 0.25. Tukey-type boxplots demonstrate median and inter-quartile level values.

Given that angiogenesis is one of the critical mechanisms of metastasis, we expected that low expressing miR-29a GC is associated with promotion of angiogenesis. GSEA revealed that the angiogenesis-related gene set was enriched in low expressing miR-29a GC (Fig. 3A; NES = − 1.79 FDR = 0.047). Additionally, most of the analyzed angiogenesis related genes, such as Angiopoietin 1 (*ANGPT1*), vascular endothelial growth factor B (*VEGFB*), and vasohibin 2 (*VASH2*), demonstrated higher expression levels in low miR-29a GC (Fig. 3A; p < 0.001, p = 0.023, and p = 0.002, respectively). These results imply that low expressing miR-29a GC is associated with enriched angiogenesis.

Since low expressing miR-29a GC enriched cell proliferation-related, as well as angiogenesis-related, gene sets we expected that cell proliferation and/or angiogenesis related genes are the top predicted target genes of miR-29a. To test this hypothesis, we utilized miRDB (http://mirdb.org), a microRNA target prediction database, to identify the top 30 target genes of miR-29a^55^. Surprisingly, only 13% of the top 30 predicted target genes were associated with cell proliferation or angiogenesis defined by the original Hallmark collection (Supplementary Figure S1). Furthermore, among the top 30 predicted miR-29a target genes, none of them were included in cell proliferation-related gene sets. These results imply that our GSEA results are not because miR-29a directly target the genes included in cell proliferation and angiogenesis gene sets, but most likely indirectly suppress these phenotypes.

### Less immune cell infiltration in low expressing miR-29a GC

**Figure 4:**
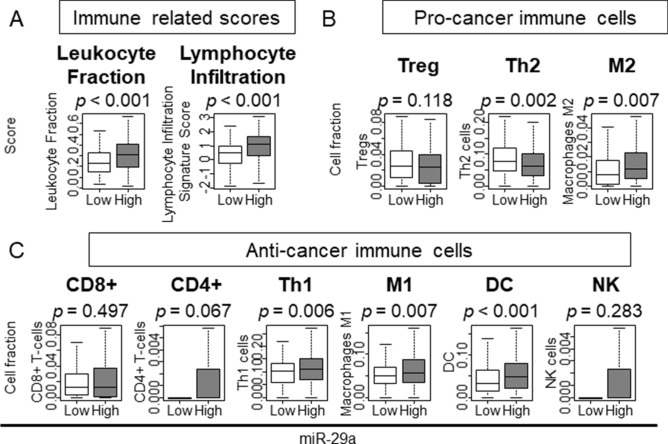
The association between miR-29a and immune related scores or infiltrated immune cells within immune tumor microenvironment (**A**) The association between miR-29a and immune related scores. (**B**) The association between miR-29a and pro-cancer immune cells. (**C**) The association between miR-29a and anti-cancer immune cells. *CD4+* CD4 positive T cell, *CD8+* CD8 positive T cell, *DC* dendritic cells, *M1/2* Macrophage M1/M2, *NK* natural killer cells, *Th1/2* type 1/2 helper T cell, *Treg* regulatory T cell; The statistical significance was defined by *p* < 0.05. Tukey-type boxplots demonstrate median and inter-quartile level values.

We previously reported that tumors with high intra-tumoral angiogenesis scores were associated with less infiltration of immune cells in breast cancer^56^. Given that angiogenesis was enriched in low expressing miR-29a GC, we expected less infiltration of immune cells in these tumors and analyzed immune-related scores utilizing previously reported datasets^57^. We found that Leukocyte Fraction score and Lymphocyte Infiltration scores were both significantly lower in low expressing miR-29a GC (Fig. 4A; p < 0.001 and p < 0.001, respectively). We then estimated the cell composition within the tumor immune microenvironment using xCell algorithm^19^. Similarly, we found lower levels of M2 macrophage (M2) in low expressing miR-29a GC, whereas T helper type 2 (Th2) cells were elevated among the pro-cancer immune cells (Fig. 4B; *p* = 0.002 and p = 0.007, respectively). T helper type 1 (Th1) cells, M1 macrophage (M1), and dendritic cell (DC) showed significantly less infiltration among the anti-cancer immune cells in miR-29a expressing GC (Fig. 4C; *p* = 0.006, *p* = 0.007 and *p* < 0.001, respectively), whereas other immune cells such CD8 T cells, CD4 T cells, and natural killer (NK) cells showed more infiltration (Fig. 4C). These results imply that immune cells in low expressing miR-29a GC infiltrate less into the tumor immune microenvironment.

### Low expressing miR-29a GC was associated with worse prognosis and low miR-29a expression was an independent predictive biomarker of worse survival. However, metastatic gastric cancer was associated with higher expression of miR-29a

**Figure 5:**
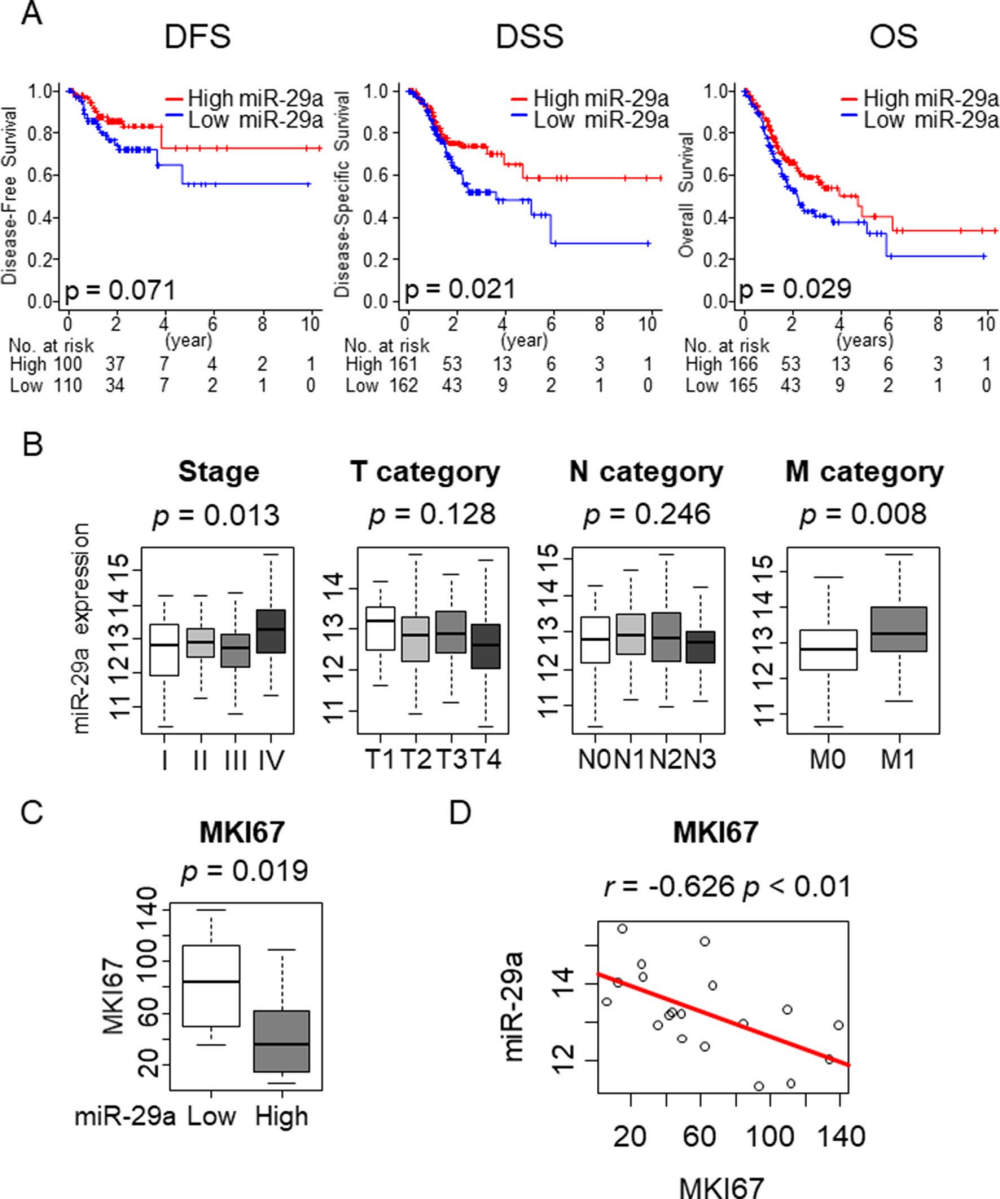
Survival analysis of miR-29a and the association between miR-29a and clinical stage in whole cohort as well as the association between miR-29a and *MKI67* expression in M1 gastric cancer patients (**A**) Kaplan Meier survival analysis of miR-29a. Blue line represents miR-29a low expressing tumors and red line represents miR-29a high expressing tumors. (**B**) The association between miR-29a and stage and TNM categories. (**C**) The association between miR-29a and *MKI67* expression in M1 gastric cancer patients. (**D**) The correlation analysis between miR-29a and *MKI67* in M1 gastric cancer patients. *DFS* disease-free survival, *DSS* disease-specific survival, *OS* overall survival. The statistical significance was defined by *p* < 0.05. Tukey-type boxplots demonstrate median and inter-quartile level values. r represents a Spearman’s correlation coefficient.

Naturally, given that low miR-29a GC is associated with cell proliferation, metastasis, angiogenesis and less anti-cancer immune cells, we chose to investigate whether it also reflects patient survival. Low expressing miR-29a GC was significantly associated with worse disease-specific survival (DSS) and overall survival (OS) (Fig. 5A; p = 0.071). There was a tendency that low expressing miR-29a GC had a worse disease-free survival; however, this was not statistically significance, which may be due to small sample size (Fig. 5A; p = 0.071). Previous reports have demonstrated miR-29a expression as a predictive biomarker for survival in gastrointestinal (GI) cancers such as hepatocellular carcinoma^58^, and gastric cancer^14,59^. To this end, we first investigated the expression levels of miR-29a in GI cancers in TCGA cohort (Supplementary Figure S2). Then, survival analysis of hepatocellular carcinoma, cholangiocarcinoma, pancreatic cancer, esophageal cancer, colon cancer, and rectal cancer were performed by high and low miR-29a expression divided by median value of each cohort. Surprisingly, there was no significant difference between the low and high expressing miR-29 tumors in any of disease-free, disease-specific, or overall survival in any of above-mentioned GI cancers in TCGA cohort (Supplementary Figure S3). To investigate whether miR-29a expression level is an independent predictive biomarker among other clinical categories, we performed Cox regression analysis. Surprisingly, along with residual tumor status, low expression of miR-29a was the only factor found to be an independent predictive biomarker in both OS and DSS (Table 1; HR = 1.50, 95% confidence interval (CI) = 1.01–2.23; p = 0.018 and HR = 1.89, 95% CI = 1.16–3.08 p = 0.010, respectively).

**Table 1:** Univariate and Multivariate analysis of miR-29a and other factors in TCGA.

Factors	Univariate		Multivariate	
	HR (95% CI)	*p* value	HR (95% CI)	*p* value
**TCGA (OS)**				
Age (≥ 65 vs < 65)	1.60 (1.09–2.34)	0.017	1.52 (1.01–2.28)	0.044
AJCC stage (III/IV vs I/II)	1.73 (1.17–2.56)	0.006	1.18 (0.62–2.27)	0.614
AJCC T factor (T3/T4 vs T1/T2)	1.64 (1.01–2.65)	0.045	1.01 (0.56–1.80)	0.983
AJCC N factor (N1/N2/N3 vs N0)	1.69 (1.08–2.65)	0.023	1.69 (0.86–3.30)	0.127
AJCC M factor (M0 vs M1)	1.17 (0.51–2.67)	0.707		
Histological grade (G3 vs G1/G2)	1.36 (0.92–2.01)	0.118		
Residual tumor (R1/R2 vs R0)	2.42 (1.35–4.35)	0.003	2.10 (1.14–3.82)	0.015
miR-29a (low vs high)	1.50 (1.04–2.18)	0.032	1.50 (1.01–2.23)	0.047
**TCGA (DSS)**				
Age (≥ 65 vs < 65)	1.18 (0.75–1.85)	0.467		
AJCC stage (III/IV vs I/II)	1.81 (1.13–2.91)	0.014	1.66 (0.96–2.90)	0.072
AJCC T factor (T3/T4 vs T1/T2)	1.82 (1.00–3.33)	0.052		
AJCC N factor (N1/N2/N3 vs N0)	1.62 (0.95–2.77)	0.080		
AJCC M factor (M0 vs M1)	1.49 (0.60–3.69)	0.391		
Histological grade (G3 vs G1/G2)	1.34 (0.84–2.13)	0.227		
Residual tumor (R1/R2 vs R0)	4.07 (2.21–7.48)	< 0.001	3.52 (1.87–6.64)	< 0.001
miR-29a (low vs high)	1.62 (1.04–2.56)	0.034	1.77 (1.08–2.95)	0.023

Given that low expressing miR-29a GC demonstrated aggressive characteristics and was associated with worse survival, we expected it would translate into more advanced clinical characteristics. Interestingly, American Joint Committee on Cancer (AJCC) Stage IV GC demonstrated higher expression levels of miR-29a compared to other stages (Fig. 5B; p = 0.013). In addition, regarding the AJCC M category, M0 gastric cancer was associated with lower expression of miR-29a (Fig. 5B; p = 0.008). However, other categories, such as T and N category, as well as pathological grade, did not demonstrate significant difference among each subcategory (Fig. 4B). Since miR-29a is a known tumor suppressive miR, we hypothesized that the elevation of miR-29a in Stage IV GC tumors is a reflection of miR-29a attempts at tumor suppression, but ultimately the aggressive nature of Stage IV GC overcomes the tumor suppressive properties of miR-29a. We found that among Stage IV GC tumors, low expressing miR-29a GC was significantly associated with high *MKI67* expression (Fig. 5C), and a strong negative correlation with *MKI67* expression (Fig. 5D; r = − 0.626, p < 0.01). Conversely there was no association between miR-29a expression and *MKI67* expression in the whole cohort. These results suggest that low expressing miR-29a GC is associated with worse survival and miR-29a expression is increased in Stage IV GC tumors, corresponding with suppression of cell proliferation.

## Discussion

In the current study, we demonstrated that low expressing miR-29a GC enriched cell proliferation and apoptosis related gene sets and was associated with high expression of apoptosis related genes, but not with pathological grade nor *MKI67* expression. Low expressing miR-29a GC enriched metastasis related gene sets and was associated with high expression of metastasis related genes. Further, low expressing miR-29a GC enriched angiogenesis related gene sets and was associated with high expression of angiogenesis related genes. We also found that low expressing miR-29a GC was associated with less leukocyte fraction and lymphocyte infiltration, as well as less anti-cancer immune cell infiltration overall. The aggressive characteristics of low expressing miR-29a GC was validated by an association with worse OS as well as DSS compared with high expressing miR-29a GC. Low miR-29a expression was the only factor, other than residual tumor status, to be an independent prognostic biomarker of worse OS and DSS. miR-29a was expressed highly in Stage IV GC tumors, but *MKI67* expression strongly inversely correlated with miR-29a expression in these tumors. The goal of this work is not to report a new mechanism of miR-29a, but to clarify the clinical relevance of miR-29a in gastric cancer utilizing a large patient cohort, TCGA, which includes both clinical and gene expression data. Although we understand the importance of delineating the novel mechanisms of a molecule, we argue studies that delineate the clinical relevance of a molecule, whether the results of in vitro studies are translatable in clinical settings, is as important a contribution toward patient care. To the best of our knowledge, the current study analyzed the role of miR-29a in the greatest number of gastric cancer patients with enrichment of multiple pathways including cell proliferation, angiogenesis stem cell markers, and immune cell infiltrations.

Previous studies have shown that miR-29a suppresses cell proliferation by targeting cell cycle related genes, such as suppressor APC domain containing 2 (*p42.3 or SAPCD2*), cyclin dependent kinase 2 (*CDK2*), cyclin dependent kinase 4 (*CDK4*), and cyclin dependent kinase 6 (*CDK6*)^16,17^. Limitations of previous studies include, data either on cell culture or animal models or very small sample size, so we investigated the clinical relevance of miR-29a expression utilizing a large GC patient cohort. We found that the previously proposed notion that miR-29a suppresses cell proliferation holds true in GC patients utilizing GSEA, which analyzes approximately 200 genes related to the pathway at one time. Utilizing this approach, we found that low expressing miR-29a GC is associated with apoptosis-related genes. Our result is in contrast to a review by Wang et al. in which they report no association between miR-29a and apoptosis in GC, even though the association was shown in other cancers such as breast cancer^60^.

Surprisingly, when looking at clinical translation in the entire GC cohort, there was no association between miR-29a and MKI67, which is the most commonly used marker of cancer cell proliferation in the clinical setting. On the contrary, significantly elevated miR-29a expression strongly inversely correlated with MKI67 expression in Stage IV GC. These results imply that miR-29a expression levels appear to correspond to tumor aggressiveness such as distant metastasis. We cannot help but speculate that the tumor suppressive function of miR-29a is clear in Stage IV GC (primary GC with distant metastasis); however, those tumors are too aggressive and overwhelms the suppressive effect of miR-29a despite being highly expressed.

Other than Nie et al. that showed miR-29a to play a role in macrophage polarization^61^, to the best of our knowledge, our study is the first to report the association between miR-29a expression and immune cell infiltration into the tumor immune microenvironment. Nie and colleagues reported that the axis of H19, a long non-coding RNA, miR-29a and collagen type I alpha 2 chain (COL1A2), may contribute to the polarization of macrophage by increasing the number of macrophage M2. We also found that M2 macrophages infiltrate significantly in high miR-29a GC but could be a result of another mechanism. We have previously published that tumors with high intra-tumoral angiogenesis were associated with immune cell infiltration as well as less immune activity^56^. The result of the current study is in the same line with this publication that low expressing miR-29a GC is associated with angiogenesis and immune cells, including Th1, M1, M2 and DC, infiltrate less.

MiR-29a expression in tumor tissue was associated with prognosis in several malignancies such as nasopharyngeal carcinoma^62^, hepatocellular carcinoma^63^, colorectal cancer^58^, and gastric cancer^14,59^. The studies by Wang et al. and Parpart et al. demonstrated that low expressing miR-29a tumors were associated with worse OS in nasopharyngeal carcinoma and hepatocellular carcinoma, respectively^62,63^. The opposite was the case in colorectal cancer patients^58^. These results imply that miR-29a may function differently depending on cancer type^58^. Regarding gastric cancer, He et al. reported that low expression of miR-29a was associated with worse OS in 58 patients^14^. Wang et al. reported similar result in a cohort of 50 patents^59^. Our study demonstrated that low expressing miR-29a GC was associated with worse survival, which was in agreement with previous studies. What is new in this study is that we used a large cohort, TCGA, and analyzed 335 patients providing more statistically reliable data. Furthermore, we have demonstrated DSS, which is more specific to the disease, excluding the possible confounding factors such as age in OS. Furthermore, we demonstrated that low expression of miR-29a is an independent prognostic biomarker in both OS and DSS.

This study is not free from limitations. First and foremost, the current study was conducted by single cohort, TCGA, which is the only one with large enough numbers of GC patients’ clinical, transcriptomic, and microRNA-sequence data. We are fully aware that findings of the current study would be more convincing if our results were validated with another cohort, however, a validation cohort was not available. Furthermore, although TCGA contains clinical data, it lacks comorbidity or therapeutic intervention data. Finally, it would be interesting to prove the mechanism of some of our novel findings by in vitro and/or in vivo experiments, such as the different roles of miR-29a in metastatic GC. However, we do not have a capability to pursue this at this time.

In conclusion, low expressing miR-29a GC was associated with aggressive cancer biology such as cell proliferation, angiogenesis, metastasis, and was associated with worse disease-free and overall survival. Low expression of miR-29a was an independent prognostic biomarker of OS and DSS in gastric cancer patients.

## Supplementary Information

Supplementary Figures.
