# Supplementary material for: Low expression of miR-29a is associated with aggressive biology and worse survival in gastric cancer
Source: Sci Rep. 2021 Jul 8;11:14134. doi: 10.1038/s41598-021-93681-z (PMC8266839; doi:10.1038/s41598-021-93681-z)
Supplement: Supplementary file 1 — Supplementary Figures. [file 41598_2021_93681_MOESM1_ESM.docx]

**Low expression of miR-29a is associated with aggressive biology and worse survival in gastric cancer**

**Yoshihisa Tokumaru^1,2^, Masanori Oshi^1,3^,** **Michelle R Huyser^1^, Li Yan^4^, Masahiro Fukada^2^, Nobuhisa Matsuhashi^2^, Manabu Futamura^2^, Yukihiro Akao^5^, Kazuhiro Yoshida^2^, Kazuaki Takabe^1,3,6-9,^***

***** Author to whom correspondence should be addressed

^1^ Breast Surgery, Department of Surgical Oncology, Roswell Park Comprehensive Cancer Center, Buffalo, NY 14263, USA;

^2^ Department of Surgical Oncology, Graduate School of Medicine, Gifu University, 1-1 Yanagido, Gifu 501-1194, Japan

^3^ Department of Gastroenterological Surgery, Yokohama City University Graduate School of Medicine, Yokohama 236-0004, Japan

^4^ Department of Biostatistics & Bioinformatics, Roswell Park Comprehensive Cancer Center, Buffalo, NY, 14263, USA

^5^ United Graduate School of Drug and Medical Information Sciences, Gifu University, 1-1 Yanagido, Gifu 501-1194, Japan

^6^ Department of Surgery, Niigata University Graduate School of Medical and Dental Sciences, Niigata 951-8510, Japan

^7^ Department of Surgery, University at Buffalo Jacobs School of Medicine and Biomedical Sciences, The State University of New York, Buffalo, NY 14263, USA

^8^ Department of Breast Oncology and Surgery, Tokyo Medical University, 6-7-1 Nishishinjuku, Shinjuku, Tokyo, 160-8402, Japan

^9^ Department of Breast Surgery, Fukushima Medical University School of Medicine, Fukushima 960-1295, Japan

**Corresponding author**

Kazuaki Takabe, MD, PhD, FACS

Breast Surgery, Department of Surgical Oncology

Roswell Park Comprehensive Cancer Center

Elm & Carlton Streets, Buffalo NY 14263 USA

Email: kazuaki.takabe@roswellpark.org

**
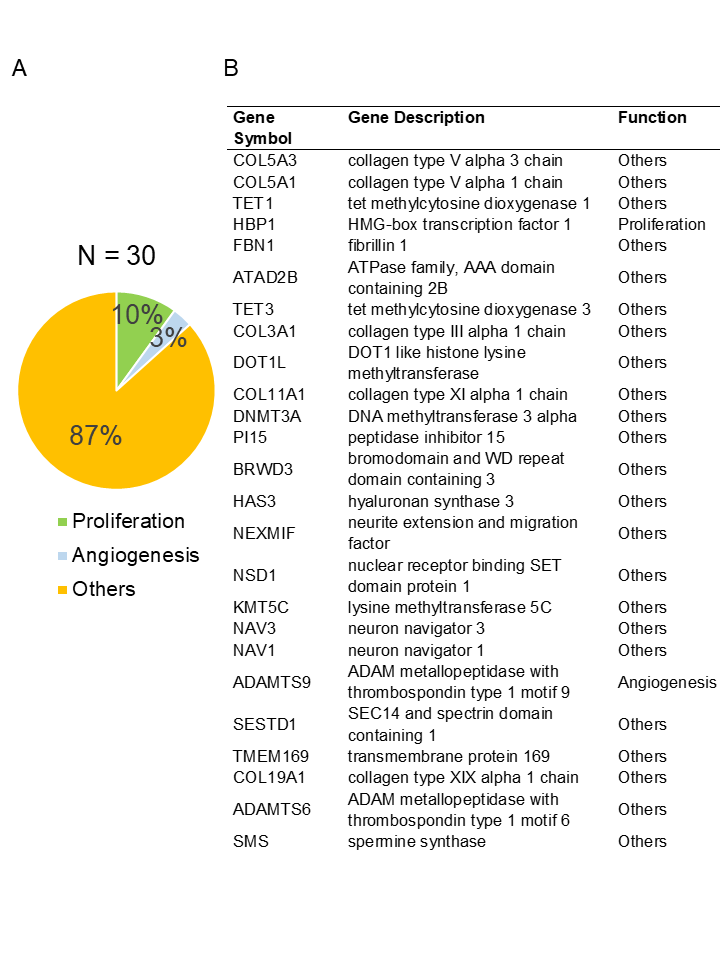
**

**Supplementary Figure S1.** Distribution and list of top 30 predicted target genes of miR-29a. (A) Distribution of predicted genes of miR-29a. (B) List of top 30 predicted target genes

**
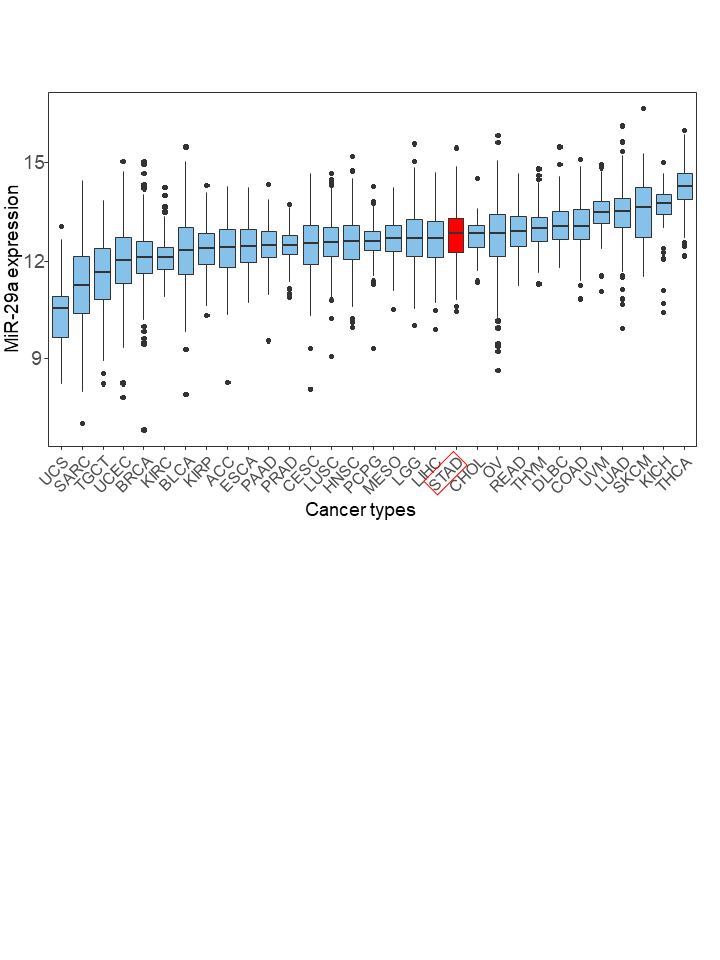
**

**Supplementary Figure S2.** The expression levels of miR-29a in various cancer types. ACC, Adrenocortical carcinoma; BLCA, Bladder Urothelial Carcinoma; BRCA, Breast invasive carcinoma; CESC, Cervical squamous cell carcinoma and endocervical adenocarcinoma; CHOL, Cholangiocarcinoma; COAD, Colon adenocarcinoma; DLBC, Lymphoid Neoplasm Diffuse Large B-cell Lymphoma; ESCA, Esophageal carcinoma; HNSC, Head and Neck squamous cell carcinoma; KICH, Kidney Chromophobe; KIRC, Kidney renal clear cell carcinoma; KIRP, Kidney renal papillary cell carcinoma; LGG, Brain Lower Grade Glioma; LIHC, Liver hepatocellular carcinoma; LUAD, Lung adenocarcinoma; LUSC, Lung squamous cell carcinoma; MESO, Mesothelioma; OV, Ovarian serous cystadenocarcinoma; PAAD, Pancreatic adenocarcinoma; PCPG, Pheochromocytoma and Paraganglioma; PRAD, Prostate adenocarcinoma; READ, Rectum adenocarcinoma; SARC, Sarcoma; SKCM, Skin Cutaneous Melanoma; STAD, Stomach adenocarcinoma; TGCT, Testicular Germ Cell Tumors; THCA, Thyroid carcinoma; THYM, Thymoma; UCEC, Uterine Corpus Endometrial Carcinoma; UCS, Uterine Carcinosarcoma; UVM, Uveal Melanoma.

**
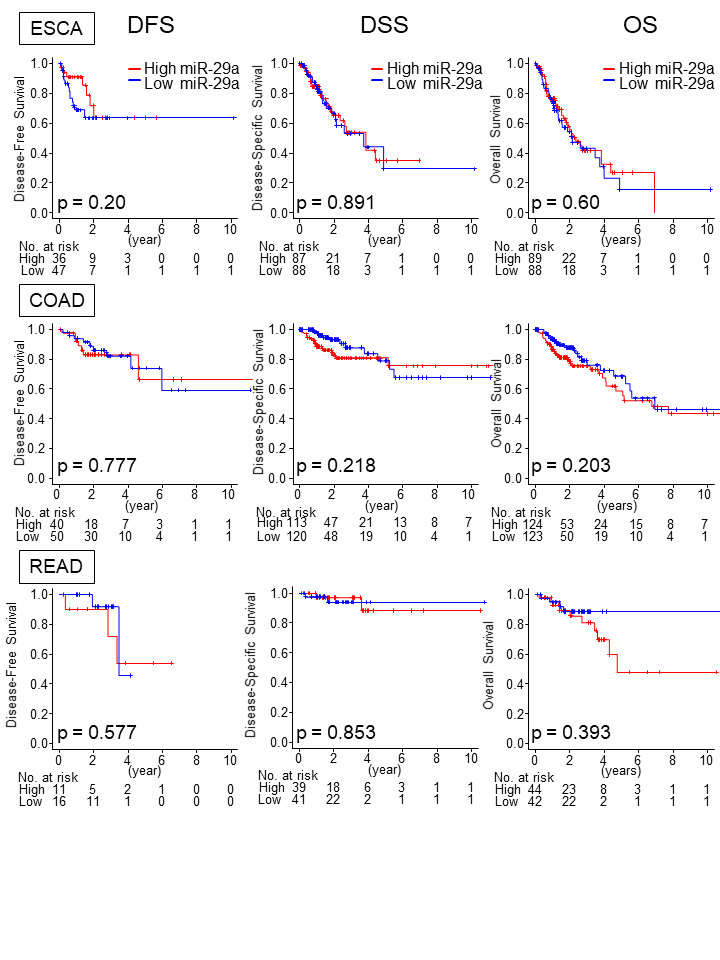
**

**
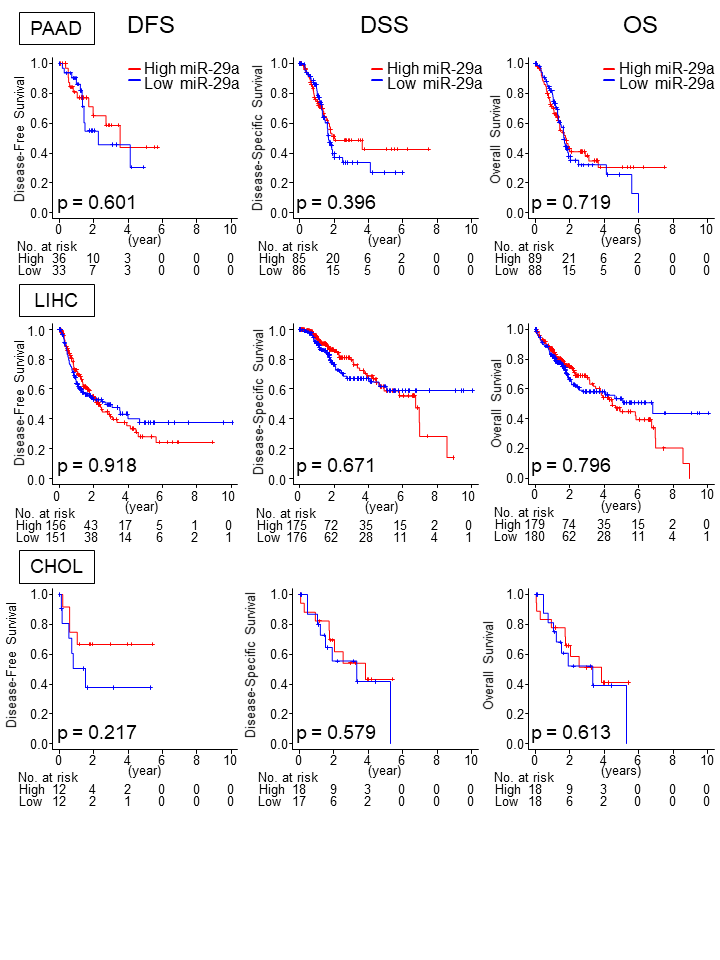
**

**Supplementary Figure S3.** Kaplan Meier survival analysis of miR-29a in gastrointestinal cancers. CHOL, Cholangiocarcinoma; COAD, Colon adenocarcinoma; ESCA, Esophageal carcinoma; LIHC, Liver hepatocellular carcinoma; PAAD, Pancreatic adenocarcinoma; READ, Rectum adenocarcinoma. The statistical significance was defined by p < 0.05.
